# Determinants of High HIV Infection Prevalence in Vijayapura, Bagalkot, and Belagavi Districts in Karnataka, India

**DOI:** 10.7759/cureus.67098

**Published:** 2024-08-17

**Authors:** Tanuja P Pattankar, Sandeep G Yankanchi, Shrinivas K Patil, Praveen R Shahapur, Chandrika R Doddihal

**Affiliations:** 1 Community Medicine, Shri B. M. Patil Medical College Hospital and Research Centre, BLDE (Deemed to be University), Vijayapura, IND; 2 Community Medicine, S. Nijalingappa Medical College and Hanagal Shree Kumareshwar (H.S.K) Hospital & Research Centre, Bagalkot, IND; 3 Microbiology, Shri B. M. Patil Medical College Hospital and Research Centre, BLDE (Deemed to be University), Vijayapura, IND

**Keywords:** sociodemographic, high burden districts, hiv aids, prevalence, people living with hiv (plhiv)

## Abstract

Introduction

India has the second-highest number of people living with human immunodeficiency virus (PLHIV). Despite the national decline in the prevalence of the human immunodeficiency virus (HIV) from 2000 to 2021, regional variations persist, particularly in the northeastern and southern states. High-risk populations, including female sex workers (FSW), men who have sex with men (MSM), and injecting drug users (IDU), significantly contribute to these dynamics. This study focuses on high-prevalence districts in Karnataka.

Objectives

This study aims to identify socioeconomic and behavioral factors associated with high HIV prevalence in high-burden districts of South Indian states.

Methodology

A cross-sectional study was conducted using data from Integrated Counseling and Testing Centers (ICTCs) and Designated STI/RTI (sexually transmitted infections/reproductive tract infections) Clinics (DSRCs) across 24 centers in the three districts. The centers were determined using a simple random sampling method. Data from 2501 HIV-positive individuals were analyzed, focusing on demographics, risk behaviors, and treatment history.

Results

Males constituted the majority of HIV cases, accounting for 448 (56.0%) in Vijayapura, 334 (51.4%) in Bagalkot, and 644 (61.1%) in Belagavi districts, with a significant portion referred by government hospitals. High HIV prevalence was linked to adults aged 25-49 years of age; the number of people with HIV was high among daily wage workers and individuals with multiple sexual partners compared to married and educated people. Newly diagnosed discordant couples ranged from 129 (12.2%) in Belagavi to 133 (18.4%) in Vijayapura districts. Most patients were on first-line antiretroviral therapy (ART), with loss to follow-up attributed to system negligence and poor compliance.

Conclusion

Key determinants of high HIV prevalence include gender, age, marital status, socioeconomic status, and sexual behavior. Effective interventions require targeted education, improved healthcare services, robust surveillance, and strengthened collaboration among stakeholders.

## Introduction

India is home to approximately 24 lakh people living with human immunodeficiency virus (HIV) infection, the second-largest country with people living with HIV (PLHIV) [1]. The national level estimated that adult HIV prevalence (15-49 years) has declined since the epidemic's peak in 2000, where prevalence was estimated from 0.55% in 2000 to 0.32% in 2010 and 0.21% in 2021. The northeastern region states have the highest adult HIV prevalence, followed by the southern states (0.67% in Andhra Pradesh, 0.47% in Telangana, and 0.46% in Karnataka). The southern states have the largest number of PLHIVs, with Maharashtra, Andhra Pradesh, and Karnataka being the top three [2]. Nevertheless, this downward trend at the national level masks the variations at the regional, state, and district levels in the country [3]. The determinant for sustained growth of PLHIV in India is through contact between high-risk populations such as female sex workers (FSW), men who have sex with men (MSM), injecting drug users (IDU), and bridge populations with forward transmission to the general population [4]. India launched the National AIDS Control Program (NACP) in 1992 for the prevention and control of HIV/AIDS. This program has a district-level focus on the implementation of prevention and control strategies based on vulnerabilities and the magnitude of the HIV burden in different districts [5]. Despite sustained efforts for HIV control for more than two decades, some districts in India are reporting consistently high HIV prevalence [2]. The factors associated with these significant and unwavering epidemics of HIV in several pockets of India are not well realized. The spread of the HIV epidemic in a defined geographic region is known to be influenced by the interaction of sociodemographic, economic, cultural, and behavioral factors [5].

Various studies conducted across the globe to understand the association of these factors with HIV have provided contrasting results. Some studies have demonstrated the association of HIV with poverty, while others have reported higher HIV levels among people from better socioeconomic strata. Although HIV is associated with illiteracy, some studies have shown a higher HIV prevalence among more educated groups. The large variations in the results of these studies highlight the fact that the findings from one country or region of the world cannot be directly extrapolated to other countries, as the factors affecting the dynamics of HIV spread vary with place and time [6-8].

There is a need to study complex inter-relationships between these socioeconomic and behavioral factors with each other and with HIV to understand the evolution and progress of the HIV epidemic in a population. Studies conducted in different parts of India have shown the association of lower literacy, higher urbanization, and socioeconomic development with higher HIV prevalence levels [9-11]. Few studies have also revealed low levels of HIV awareness and condom use in India [12].

## Materials and methods

After securing institutional ethical clearance (reference number BLDE(DU)/IEC/846/2022-23), this study was conducted in Integrated Counseling and Testing Centers (ICTCs) and Designated STI/RTI Clinics (DSRCs) within the Vijayapura, Bagalkot, and Belgaum districts of North Karnataka, areas recognized for their high HIV incidence rates. These districts house 22, 17, and 35 ICTC centers, respectively. A simple random sampling method was employed to select 30% of these centers, resulting in a total of 24 centers: seven from Vijayapura, six from Bagalkot, and 11 from Belgaum. This selection particularly targeted centers with high client loads to ensure the study's relevance and accuracy. Furthermore, all three DSRCs, one in each district, were included to provide a comprehensive data set.

The study focused on analyzing records from HIV-positive individuals registered at these centers over the past three years. Data collection covered a wide range of demographic and behavioral factors, including age, gender, referral source, education level, occupation, marital status, type of risk behavior, consent for HIV testing, test results, follow-up records, referral information, partner's HIV status, and condom usage. This comprehensive approach allowed for a detailed examination of the factors contributing to the high HIV prevalence [5].

By reviewing and analyzing these variables, the study aimed to identify key patterns and associations that could inform future public health strategies and interventions. The ultimate goal was to enhance understanding of the epidemiological dynamics of HIV in these high-burden districts and to support the development of targeted efforts to reduce HIV transmission and improve patient outcomes.

Statistical analysis

Data were entered into a Microsoft Excel sheet (Microsoft Corporation, Redmond, Washington) and analyzed using IBM SPSS Statistics for Windows, Version 20 (Released 2011; IBM Corp., Armonk, New York). The results were presented as mean ± SD, counts, percentages, and diagrams. Categorical variables were compared using statistical tests such as the chi-square test, with a significance level set at p < 0.05.

## Results

The study yielded a total of 2501 PLHIV, obtained from the three-year records of the ICTC and DSRC centers in the three districts. Table 1 compares demographic variables across Vijayapura, Bagalkot, and Belagavi, highlighting significant differences in gender and age distributions. Vijayapura has a higher percentage of males (448, 56.0%) compared to Bagalkot (334, 51.5%) and Belagavi (644, 61.1%), which was statistically significant at p < 0.05. Age distribution also shows significant variation, with Belagavi having a higher percentage of individuals aged 25-34 years old (311, 29.5%) compared to Vijayapura (189, 23.6%) and Bagalkot (131, 20.2%). Marital status does not significantly differ across the regions (p = 0.226), indicating similar proportions of married, single, divorced/separated, and widowed individuals.

**Table 1:** Sociodemographic profile of PLHIV in the three districts *p < 0.05 was considered statistically significant PLHIV: people living with human immunodeficiency virus

Variables	Vijayapura (799)		Bagalkot (648)		Belagavi (1054)		Value of Chi-Square Test	P-value
	N	%	N	%	N	%		
Gender								
Male	448	56.0	334	51.5	644	61.1	15.39	<0.00045*
Female	351	44.0	314	48.5	410	38.9		
Age in completed years								
<14	37	4.6	35	5.4	41	3.9	63.22	<0.0001*
15–24	100	12.5	141	21.7	198	18.8		
25–34	189	23.6	131	20.2	311	29.5		
35–49	335	41.9	203	31.3	305	28.9		
>50	138	17.3	138	21.3	199	18.9		
Marital status								
Married	238	29.8	212	32.7	294	27.9	8.22	0.226
Single	275	34.4	202	31.2	338	32.1		
Divorce/separated	158	19.8	131	20.2	234	22.2		
Widowed	120	15.0	103	15.9	188	17.8		

Table 2 compares the socioeconomic status of PLHIV using educational and occupational distributions as a proxy across the three high-burden districts. Education levels vary notably (p < 0.0001), with higher illiteracy rates in Belagavi (135, 12.8%) compared to Vijayapura (49, 6.1%) and Bagalkot (65, 10.0%). Primary and secondary education distributions also differ, with Belagavi having the highest primary education rate (557, 52.8%) and the lowest secondary education rate (212, 20.1%). Occupation distributions also show significant variation (p < 0.0001), with daily wage earners being most prevalent in Vijayapura (473, 59.2%) and least in Belagavi (508, 48.3%). Belagavi has a higher percentage of salaried individuals (295, 28.1%) compared to Vijayapura (184, 23.0%) and Bagalkot (122, 18.8%).

**Table 2:** Socioeconomic determinants of PLHIV in the three districts *p < 0.05 was considered statistically significant PLHIV: people living with human immunodeficiency virus

Variables	Vijayapura (799)		Bagalkot (648)		Belagavi (1054)		Value of Chi-Square Test	P-value
	N	%	N	%	N	%		
Education								
Illiterate	49	6.1	65	10.0	135	12.8	108.63	<0.0001*
Primary school	352	44.0	303	46.7	557	52.8		
Secondary school	321	40.2	225	34.7	212	20.1		
College & above	77	10.7	55	8.5	150	14.2		
Occupation								
Daily wages	473	59.2	364	56.2	508	48.3	40.33	<0.0001*
Salaried	184	23.0	122	18.8	296	28.1		
Business	64	8.0	85	13.1	108	10.2		
Housewife	78	9.8	77	11.9	142	13.4		

Table 3 examines the distribution of behavioral factors associated with PLHIV in the three districts. A higher percentage of individuals in Belagavi (965, 91.6%) reported multiple heterosexual partners compared to Vijayapura (712, 89.1%) and Bagalkot (559, 86.3%) (p < 0.005). In Vijayapura, 133 (16.6%) of couples have recently been found to have a discordant HIV status (one partner positive) among spouses/partners of HIV-positive individuals, compared to 119 (18.4%) in Bagalkot and 129 (12.2%) in Belagavi. Couples where the husband is negative and the wife is positive are highest in Bagalkot (255, 39.4%) and lowest in Belagavi (355, 33.7%). Conversely, couples where the husband is positive and the wife is negative are highest in Belagavi (415, 39.4%) and lowest in Bagalkot (218, 33.6%). Newly detected concordant positive couples are most common in Belagavi (155, 14.7%) and least common in Vijayapura (63, 7.9%). These variations highlight regional differences in HIV transmission among couples (Table 3).

**Table 3:** Behavioral factors associated with PLHIV in the three districts *p < 0.05 was considered statistically significant PLHIV: people living with human immunodeficiency virus

Variables	Vijayapura (799)		Bagalkot (648)		Belagavi (1054)		Value of Chi-Square Test	P-value
	N	%	N	%	N	%		
Multiple sexual partners (heterosexual)	712	89.1	559	86.3	965	91.6	19.14	<0.005*
Multiple sexual partners (homosexual/bisexual)	69	8.6	66	10.2	54	5.1		
Transmitted from parent to child (for children)	18	2.3	23	3.5	35	3.3		
HIV status of the spouse/partner of HIV-positive person								
Number of newly detected discordant couples/partners	133	16.6	119	18.4	129	12.2	42.52	<0.0001*
Number of couples where the husband/male partner is negative and the wife/female partner positive	298	37.3	255	39.4	355	33.7		
Number of couples where the husband/male partner is positive and the wife/female negative	305	38.2	218	33.6	415	39.4		
Number of newly defected concordant couples (both positive)	63	7.9	56	8.6	155	14.7		

**Figure 1:**
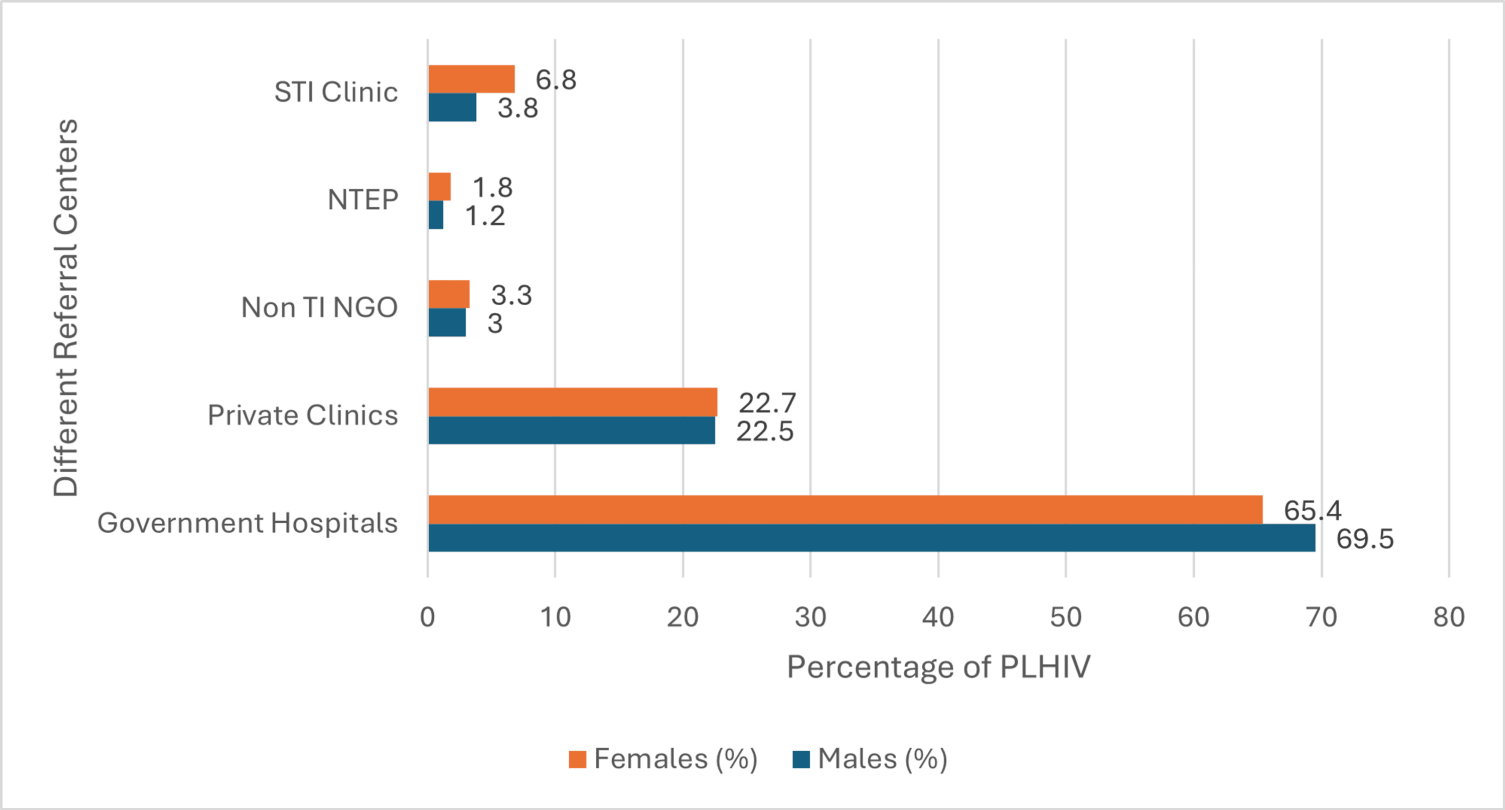
Gender-specific distribution of PLHIV referrals from the three districts The total N is 2501, with 1426 males and 1075 females STI: sexually transmitted infection; NTEP: National Tuberculosis Elimination Programme; non-TI NGOs: non-targeted intervention NGOs: PLHIV: people living with human immunodeficiency virus

The bar chart in Figure 1 illustrates the gender-wise distribution of PLHIV referrals from various healthcare centers across the three high-burden districts in Karnataka. Government hospitals are the primary referral source for both genders, accounting for 991 (69.5%) of male and 703 (65.4%) of female referrals. Private clinics are the second most common referral source, with nearly equal percentages for males 321 (22.5%) and females 244 (22.7%). Referrals from sexually transmitted infection clinics (STI clinics), the National Tuberculosis Elimination Programme (NTEP), and non-targeted intervention NGOs (non-TI NGOs) were minimal.

The treatment history revealed that a large majority, i.e., 1355 (95%) of males and 1042 (96.9%) of females, were on first-line antiretroviral therapy (ART), while a smaller proportion of males (71, 5%) and females (34, 3.1%) required second-line ART. Loss to follow-up accounted for 37 (1.47%) of the total PLHIV of 2501 samples, which is attributed to system negligence, poor patient knowledge, poor compliance, and drug side effects.

## Discussion

This study provides a comprehensive analysis of the demographic, socioeconomic, and behavioral profiles of PLHIV across three high-burden districts in Karnataka: Vijayapura, Bagalkot, and Belagavi. The total sample size, derived from three years of records at ICTC and DSRC, includes 2501 individuals, offering valuable insights into the patterns and factors associated with HIV transmission and care.

The demographic analysis reveals significant differences across the districts. Vijayapura has a higher percentage of males (56.0%) compared to Bagalkot (51.5%) and Belagavi (61.1%), with a statistically significant p-value of <0.05. This gender disparity highlights potential regional differences in the detection and reporting of HIV cases among men, possibly linked to health-seeking behavior or occupational exposure [13]. Age distribution also varies significantly, with Belagavi showing a higher percentage of individuals aged 25-34 years (29.5%) compared to Vijayapura (23.6%) and Bagalkot (20.2%). This age group represents a critical segment for targeted interventions due to their high mobility and sexual activity, increasing their risk of HIV transmission [14]. Marital status, however, does not show significant differences across the regions, indicating similar proportions of married, single, divorced/separated, and widowed individuals.

Education and occupation distributions highlight notable socioeconomic disparities. Belagavi has the highest illiteracy rate (12.8%) compared to Vijayapura (6.1%) and Bagalkot (10.0%), with a significant p-value of <0.0001. Additionally, Belagavi shows the highest primary education rate (52.8%) and the lowest secondary education rate (20.1%), suggesting a need for improved educational outreach and support to enhance health literacy among PLHIV [15]. Occupation-wise, daily wage earners are most prevalent in Vijayapura (59.2%) and least in Belagavi (48.3%). However, Belagavi has a higher percentage of salaried individuals (28.1%) compared to Vijayapura (23.0%) and Bagalkot (18.8%), indicating better economic stability among some segments of the population. These socioeconomic factors are crucial for understanding the accessibility and adherence to HIV treatment and care [16].

Behavioral factors associated with HIV transmission show significant regional differences. A higher percentage of individuals in Belagavi (91.6%) report multiple heterosexual partners compared to Vijayapura (89.1%) and Bagalkot (86.3%), with a p-value of <0.005. This underscores the need for targeted behavioral interventions and education to reduce risky sexual behaviors and prevent new infections [17]. The distribution of newly detected discordant couples is higher in Bagalkot (18.4%) compared to Vijayapura (16.6%) and Belagavi (12.2%), with a highly significant p-value of <0.0001. This indicates varying levels of HIV awareness and testing among couples in different districts. Moreover, Belagavi has a higher rate of concordant positive couples (14.7%) compared to Vijayapura (7.9%) and Bagalkot (8.6%), highlighting regional differences in the dynamics of HIV transmission within couples [13].

Figure 2 shows that government hospitals are the primary referral source for both genders, accounting for 69.5% of male and 65.4% of female referrals. Private clinics are the second most common referral source, with nearly equal percentages for males (22.5%) and females (22.7%). Referrals from STI clinics, NTEP, and non-TI NGOs are minimal, suggesting that these facilities play a limited role in the referral network. Strengthening the integration of these services could enhance the overall referral system and improve access to HIV care [18].

The majority of PLHIV are on first-line ART, with 95% of males and 96.9% of females receiving this treatment. A smaller proportion (5% males, 3.1% females) require second-line ART, indicating good adherence and effectiveness of the first-line regimen for most patients. However, the loss to follow-up rate of 1.47%, attributed to system negligence, poor patient knowledge, compliance issues, and drug side effects, highlights the need for improved patient education and support systems to ensure continuous care [5].

The main limitation of this study is that it was conducted in a single district in southern India, which may restrict the generalizability of the findings. Conducting a multicentric study with a larger sample size would yield more robust results, allowing for better extrapolation and a more comprehensive analysis of the factors influencing HIV infection across different population groups in India. Additionally, another limitation is that the data were obtained in consolidated numbers from various centers over the previous two years. Hence, regression models could not be applied in this study.

## Conclusions

The study provided a comprehensive analysis of the social, economic, and behavioral factors influencing HIV/AIDS incidence and prevalence in Bagalkot, Vijayapura, and Belagavi districts over the past three years. Key findings emphasized the impact of gender, age, marital status, socioeconomic status, and sexual behavior on transmission dynamics. Notably, adults aged 25 to 49, particularly daily wage workers, were most affected. Addressing risky sexual behaviors, especially multiple partnerships, is critical. Vigilant monitoring and targeted interventions are essential to sustain progress, requiring strengthened healthcare systems and enhanced collaboration among stakeholders.
